# A Single-Carbon Stable Isotope Ratio Model Prediction Equation Can Estimate Self-Reported Added Sugars Intake in an Adult Population Living in Southwest Virginia

**DOI:** 10.3390/nu13113842

**Published:** 2021-10-28

**Authors:** Valisa E. Hedrick, Tanya M. Halliday, Brenda M. Davy, Jamie M. Zoellner, A. Hope Jahren

**Affiliations:** 1 Department of Human Nutrition, Foods, and Exercise, Virginia Tech, Blacksburg, VA 24061, USA; bdavy@vt.edu; 2 Department of Health, Kinesiology, and Recreation, University of Utah, Salt Lake City, UT 84112, USA; tanya.halliday@utah.edu; 3 Department of Public Health Sciences, University of Virginia, Christiansburg, VA 24073, USA; jz9q@eservices.virginia.edu; 4 Centre for Earth Evolution and Dynamics, University of Oslo, 0371 Oslo, Norway; a.h.jahren@geo.uio.no

**Keywords:** biomarker, added sugars, dietary assessment, carbon stable isotopes

## Abstract

The δ^13^C value of blood is a novel proposed biomarker of added sugars (AS) intake. AS prediction equations using either a single- (δ^13^C) or dual-isotope model (δ^13^C and δ^15^N) were previously developed in an adult population with high AS intake living in southwest Virginia (reference group). The purpose of this investigation was to test the δ^13^C single- and δ^13^C and δ^15^N dual-isotope prediction equations for AS intake in adults with a lower mean AS intake and different demographic characteristics (test group). The blood samples for the reference (*n* = 257 for single-isotope, *n* = 115 for dual-isotope) and test groups (*n* = 56) were analyzed for δ^13^C and δ^15^N values using natural abundance stable isotope mass spectrometry and were compared to reported dietary AS intake. When the δ^13^C single-isotope equation was applied to the test group, predicted AS intake was not significantly different from reported AS intake (mean difference ± standard error = −3.6 ± 5.5 g, Z = −0.55, *p* = 0.51). When testing the dual-isotope equation, predicted AS was different from reported AS intake (mean difference ± SEM = 13.0 ± 5.4 g, Z = −2.95, *p* = 0.003). δ^13^C value was able to predict AS intake using a blood sample within this population subset. The single-isotope prediction equation may be an alternative method to assess AS intake and is more objective, cost-feasible, and efficient than traditional dietary assessment methods. However, more research is needed to assess this biomarker with rigorous study designs such as controlled feeding.

## 1. Introduction

The most commonly cited limitation of most dietary assessment methods is the error associated with the use of self-reported dietary intake from dietary intake recalls/records and food-frequency questionnaires [1,2]. Under-reporting is especially prevalent when assessing intake of dietary items considered unhealthy such as added sugars (AS) and sugar-sweetened beverages [2]. As many public policies have been suggested regarding AS and sugar-sweetened beverages [3], the ability to accurately assess specific dietary intake is needed. Dietary biomarkers that objectively measure dietary intake can help to overcome these limitations [2,4]. The availability of dietary biomarkers may improve assessments of public policy impact on specific dietary consumption. Furthermore, implementing dietary biomarkers into national surveillance data collection (e.g., National Health and Nutrition Examination Survey; NHANES) could provide dietary intake trends and correlations with health status.

One such biomarker is the δ^13^C value of blood [5]. δ^13^C is a novel proposed biomarker of AS intake that increases with consumption of C4 plants (corn [e.g., high-fructose corn syrup] and cane sugars), which exhibit high δ^13^C values [6]. The δ^13^C biomarker has established validity in several clinical laboratory-based investigations and within community-based settings, which have explored different geographical populations and tissue assay samples [5,7,8,9,10]. The majority of δ^13^C biomarker studies have examined either the potential of δ^13^C as a dietary biomarker and/or the preliminary comparative validity of habitual measures of δ^13^C (i.e., tissue samples with longer turnover times) through cross-sectional investigations using 24 h dietary recalls, dietary records, and food-frequency questionnaires for adults [7,10,11] and record-assisted 24 h dietary recalls for children and adolescents [12]. Despite the reported correlations for this research area (*R*^2^ range = 0.03–0.33) [7,10,11,12] being on the lower end of the spectrum of typical correlations reported for dietary biomarker validation studies (*R*^2^ range = 0.02–0.93) [4], the associations between δ^13^C and AS intake have remained consistent across various investigations for specific geographical locations [7,10,11,12]. Feeding studies may produce improved *R*^2^ values over self-reported studies due to the potential for under-reporting consumption of socially undesirable foods such as sugar-sweetened beverages or sweets [13,14]. A randomized controlled trial aimed at reducing sugar-sweetened beverage intake has demonstrated the ability of the δ^13^C biomarker to be sensitive to changes in AS consumption as compared to reported 24 h dietary recalls [8]. In addition, one small (*n* = 5) feeding study has demonstrated the capability of the δ^13^C biomarker as a potential AS biomarker [15]. However, the tissue sample assessed during the feeding study (i.e., δ^13^C of glucose) was a dynamic assay with a short turnover time; thus, comparison of the validity of the results was not feasible.

Despite these studies demonstrating the possibility of a valid biomarker, it is important to note that this biomarker is most appropriate for populations that primarily use C4 sugar sources (e.g., United States) and may not accurately represent AS intake in populations that use sugar beets (C3 plant) as a primary sweetener source (e.g., European countries) [16]. Furthermore, populations with a high consumption of marine animals or animal protein with corn as a primary food source (i.e., consumption of δ^13^C “enriched” protein) may demonstrate elevated δ^13^C values not resulting from AS consumption [17]. As some animal protein sources demonstrate elevated δ^15^N levels compared to plant products, δ^15^N may be used as a “correction factor” to account for the impact of consumption on δ^13^C levels [10,16,18]. However, results are mixed if a dual-isotope model (δ^13^C and δ^15^N) demonstrates improved prediction of AS intake over a single-isotope model (δ^13^C), with most varying results occurring between populations with a different underlying isotopic ecology of the diet [9]. Previous research has shown that the dual model does not provide additional AS consumption predictive ability over the single-isotope model for a population living in southwest Virginia [9]. For example, investigations that used individuals from southwest Virginia have demonstrated an *R*^2^ of 0.08–0.14 between a single-isotope model δ^13^C and AS intake [7,9,12] and no additional benefit of including δ^15^N values (*R*^2^ = 0.11) [9]. In contrast, for a Yup’ik population residing in Alaska with a high consumption of marine animal protein, prediction of AS intake improved when a dual-isotope model was utilized (single-isotope *R*^2^ = 0.03 vs. dual-isotope *R*^2^ = 0.33) [10].

AS prediction equations (single- and dual-isotope models) were previously developed using fingerstick blood samples from an adult population with a high reported AS intake residing in southwest Virginia, with findings suggesting that a δ^13^C single-isotope model may be a superior objective measure of AS consumption compared to a dual-isotope model [9]. The objective of this investigation was to further examine the potential of these equations to predict AS intake in a different adult population with a lower reported mean AS intake and varying tissue assay samples (i.e., serum and plasma).

## 2. Materials and Methods

### 2.1. Subjects and Design

Reference group: The AS single- and dual-isotope prediction equations were previously developed and published using baseline data from participants who were enrolled in a sugar-sweetened beverage reduction intervention trial, i.e., the “reference group” [9]. The reference prediction equations were established using fingerstick δ^13^C (mean ± SD = −19.1 ± 0.8‰) and δ^15^N (mean ± SD = 7.4 ± 0.5‰) blood samples from an adult population (mean age ± SD = 42 ± 15 years) with high AS intake (mean daily intake ± SD = 89 ± 59 g; median daily intake = 70.9 g) residing in southwest Virginia [9]. The single- and dual-isotope model prediction equations were developed from 257 and 115 participants’ data, respectively.

The first equation utilized a single-carbon stable isotope ratio model (δ^13^C): ln(Predicted added sugars intake) = 8.01 + 0.19 (δ^13^C) − 0.004 (age). The second equation utilized a dual-carbon-and-nitrogen stable isotope ratio model (δ^13^C and δ^15^N): ln(Predicted added sugars intake) = 5.50 + 0.13 (δ^13^C) + 0.18 (δ^15^N) − 0.004 (age).

Test group: The next step was to apply these equations in a different adult population (i.e., the test group) to determine their abilities to be valid predictors of AS intake. This investigation (total: *n* = 56) utilized participant baseline data compiled from three previous trials [19,20,21]. The purpose of the first study was to examine the impact of weight loss on arterial destiffening and included 30 adults with overweight/obesity aged 55–75 years old [19]. The purpose of the second study was to examine the impact of weight gain on arterial destiffening and included 11 men without obesity aged 18–26 years old [21]. The purpose of the third study was to examine the effectiveness of atorvastatin treatment to reduce arterial stiffness and included 15 adults with overweight/obesity aged 40–65 years old [20]. Participants with measured δ^13^C and δ^15^N values and AS consumption via self-reported dietary intake were selected from these trials to be included in this analysis to validate the previously developed equations. It is important to note that regardless of participant study enrollment, only baseline δ^13^C and δ^15^N values were used for this analysis; thus, study interventions had no influence on these findings.

### 2.2. Ethics

This study was conducted according to the guidelines in the Declaration of Helsinki, and the Virginia Tech Institutional Review Board approved the study protocol. Participants provided written informed consent prior to enrollment.

### 2.3. Measures

Anthropometrics: Participants in both the reference and test groups underwent assessments of height, which was measured in meters without shoes using a stadiometer, weight, which was measured wearing light clothing without shoes to the nearest 0.1 kg using a digital scale, and calculated body mass index (BMI) (kg/m^2^).

Dietary intake: For the reference group [22], estimated dietary intake, including AS intake, was assessed using three self-reported 24 h dietary recalls collected on non-consecutive days. The first dietary recall was completed in-person, and the following two recalls were completed via unannounced telephone calls within a two-week period. One weekend and two weekdays were recalled using the multiple pass method. Trained research technicians who were supervised by a doctoral-level registered dietitian nutritionist collected the recalls. For the present test group, estimated dietary intake was assessed via self-reported four-day food intake records collected at baseline. Participants recorded their intake either Wednesday through Saturday or Sunday through Wednesday to reflect intake on weekends and weekdays. Records were reviewed with participants for accuracy, completeness, and clarity. Both the recalls and records for the reference and test groups were analyzed with Nutrition Data System for Research (NDSR 2011) nutritional analysis software [23].

Isotope analysis: For the reference group, the participants provided whole blood samples via a routine fingerstick and blotted onto sterilized Whatman spun-glass filters (type GF/D, 2.5 cm; GE Healthcare). Punches, 3.1 mm in diameter, were collected from air-dried samples and loaded into high-purity tin capsules. Using natural abundance stable isotope mass spectrometry, samples were quantitatively combusted to carbon dioxide in a Costech ECS 4010 Elemental Analyzer (Costech Analytical) coupled to a Delta V Advantage Isotope Ratio Mass Spectrometer (Thermo Fisher). For the test group, participants provided fasting blood samples (samples were either serum or plasma, distinction was not recorded) which were collected via venipuncture at baseline. Serum was collected in silicone-coated BD vacutainers, allowed to clot at room temperature for 15 min, and centrifuged at 4 °C for 15 min at 2500× *g*. Plasma was collected in K3 EDTA BD vacutainers and was immediately centrifuged at 4 °C for 15 min at 2500× *g*. Samples were then combusted to carbon dioxide using the above stated procedure. The stable isotope values are reported in standard δ-notation: δ = R_sample_/R_standard_ − 1, where R is the isotope ratio (^13^C/^12^C or ^15^N/^14^) in the sample or international standard (Vienna Pee Dee Belemnite; VPDB) for carbon, atmospheric AIR for nitrogen). δ^13^C and δ^15^N values are reported in units of permil (‰) and represent the mean of three analyses. L-alanine was used as an internal laboratory standard for both δ^13^C and δ^15^N, which was previously calibrated against the International Atomic Agency standards NBS-19 limestone and L-SVEC lithium carbonate for δ^13^C and against USGS-40 and 41 glutamic acid for δ^15^N. The total range across the three measurements of each sample never exceeded 0.1‰. An analytical uncertainty of <±0.1‰ was associated with each sample measurement, resulting in an intra-assay coefficient of variation of 0.1‰ [7,9]. Blood samples for both the reference and test groups were collected on the same day as the first dietary recall or record.

### 2.4. Data Analysis

Statistical analyses were performed using SPSS statistical analysis software (version 24.0 for Windows, 2016; IBM). Descriptive statistics (means ± standard deviation and frequencies) were reported for demographic characteristics, as well as χ^2^ and one-factor ANOVA tests to examine potential demographic and dietary intake differences between the reference and test groups. The single- and dual-isotope prediction equations from the reference group were applied to this test group’s data. As the groups displayed non-normal data for δ^13^C values and reported AS intake, a Wilcoxon signed ranks test and Spearman’s rho correlation were used to compare reported versus predicted AS intake. Bland–Altman analyses were used to compare reported versus predicted AS intake at an individual level [24,25,26,27]. Bland–Altman plots recommend that 95% of the data points are within ±1.96 standard deviations of the mean difference, and the mean difference should be close to 0. The statistical limits were calculated from the mean and standard deviation of the difference between the two AS measurement methods (reported vs. predicted AS intake) [24,25,26,27]. The significance level was set a priori at *p* ≤ 0.05.

## 3. Results

### 3.1. Demographics

Compared to the reference group, the test group was significantly (*p* ≤ 0.001) older with a greater proportion of males, but BMI was not significantly different. In addition, this test group had significantly lower δ^13^C (*p* ≤ 0.01) values and greater δ^15^N (*p* ≤ 0.001) values. δ^13^C values ranged from −22.8 to −17.5‰ with a median value of −19.4‰ and δ^15^N values ranged from 8.3 to 9.8‰ with a median value of 9.1‰. This test group consumed less mean AS g (*p* ≤ 0.05) and had a lower median intake (test group median = 59.6 g, reference group median = 70.9 g). The test group’s AS intake ranged from 5.7 to 212.9 g and the reference group’s AS intake ranged from 5.4 to 329.8 g (see Table 1).

### 3.2. Single-Isotope Equation

**Figure 1:**
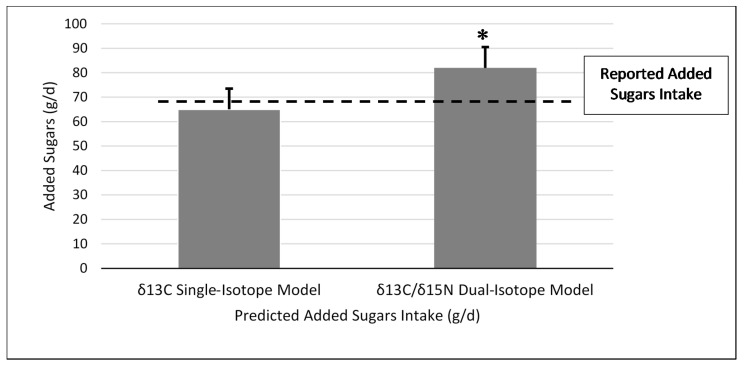
Reported added sugars intake compared to predicted added sugars intake for the δ^13^C single-carbon and δ^13^C and δ^15^N dual-carbon and nitrogen stable isotope ratio prediction models in the test group (*n* = 56)**.** * Significant difference between reported added sugars intake and dual-isotope model at *p* = 0.02. δ^13^C and δ^15^N dual-isotope model (mean difference ± standard error = 13.0 ± 5.4 g; Z = −2.95; *p* = 0.003). No significant difference was found between reported added sugars intake and predicted added sugars intake for the δ^13^C single-isotope model (mean difference ± standard error = −3.6 ± 5.5 g; Z = −0.55, *p* = 0.59).

**Figure 2:**
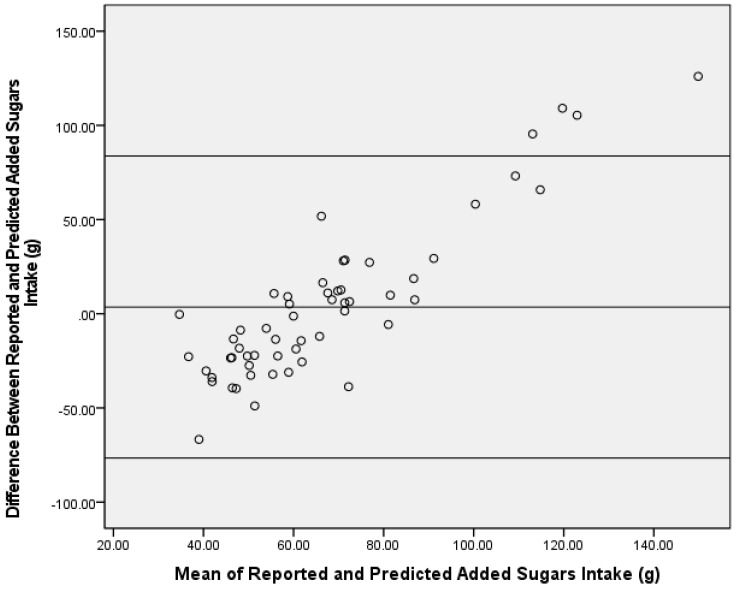
Bland–Altman analysis of reported and predicted added sugars intake (g) using a δ^13^C single-carbon stable isotope ratio prediction model in the test group (*n* = 56)**.** The center line represents the mean difference, and the upper and lower lines indicate the mean ± 1.96 standard deviation.

Using the δ^13^C single-isotope equation, predicted AS intake was not significantly different from reported AS intake in the test group (mean difference ± SEM = −3.6 ± 5.5 g; Z = −0.55, *p* = 0.59) (Figure 1), and predicted and reported AS intake was significantly correlated (ρ = 0.34; *p* = 0.01). Bland–Altman analysis did not demonstrate acceptable agreement between reported AS intake from the dietary recalls and the single-isotope model (93%) (Figure 2).

### 3.3. Dual-Isotope Equation

**Figure 3:**
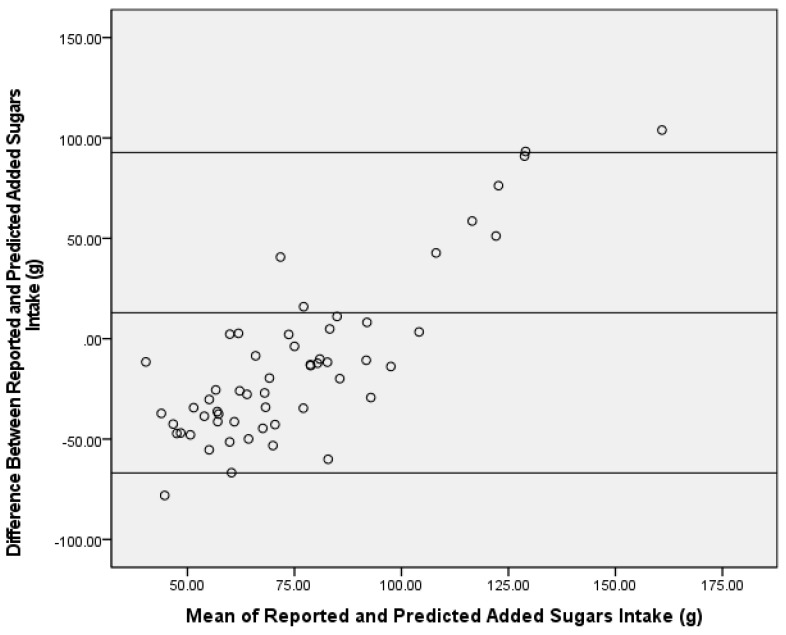
Bland–Altman analysis of reported and predicted added sugars intake (g) using a δ^13^C and δ^15^N dual-carbon stable isotope ratio prediction model in the test group (*n* = 56)**.** The center line represents the mean difference, and the upper and lower lines indicate the mean ± 1.96 standard deviation.

When applying the δ^13^C and δ^15^N dual-isotope equation to the test group, predicted AS was significantly greater than reported AS intake (mean difference ± SEM = 13.0 ± 5.4 g, Z = −2.95, *p* = 0.003) (Figure 1), and the correlation was similar to the single-isotope model (ρ = 0.40, *p* = 0.002). In addition, the Bland–Altman analysis did not demonstrate agreement between reported AS intake from the dietary recalls and the dual-isotope model (91%) (Figure 3).

## 4. Discussion

These findings demonstrate the potential to predict AS intake in this population subset with the use of a prediction equation with the δ^13^C value of human blood and age as variables. Specifically, this prediction equation was confirmed in a test population with a lower AS intake and with varying δ^13^C specimen types (i.e., serum and plasma vs. fingerstick blood samples). Similar to other investigations, these results suggest that various blood specimen types can be used to measure δ^13^C values, thereby increasing the utility of the biomarker [5,18,28]. For example, Nash et al., compared stable isotope values across various specimens including hair, plasma, and red blood cells and concluded that stable isotope values can be compared across specimen types [29]. Further, they suggested that these findings, along with Kraft et al., [30] demonstrate the ability of serum and plasma stable isotope values to be analogous across studies.

Despite the associations found between δ^13^C values and AS intake for this study population being lower than reported correlations for other dietary biomarker validation studies [4], these correlations for predicting AS intake were similar to previous δ^13^C work [7,9,12]. The reported correlations were not directly comparable to previous literature, as this investigation examined the correlation between predicted and reported AS intake (versus δ^13^C value and AS intake). However, predicted AS intake is a function of δ^13^C values from the prediction equations, hence, similar correlations. Typically, a Bland–Altman plot demonstrating ≥95% consensus is considered acceptable agreement between assessment tools [24,25,26,27]. Bland–Altman analysis results for the δ^13^C single-isotope equation fell slightly below this standard at 93% between reported and predicted AS intake. When examining the Bland–Altman plot for direction of disagreement, the number of predictions that were over- versus under-reported were similar with a little over half (55%) of the samples’ predicted AS intake being over-reported by the equation and 45% being under-reported. Despite the similar distribution for over- and under-reported AS intake, the Bland–Altman plots suggest proportional bias towards those with higher AS intake. Upon further investigation, the four participants with a mean difference of AS intake between reported and predicted AS intake above the upper limit were the four highest consumers of reported AS as well as the four highest predicted AS consumers. In addition, these four participants were the only outliers based on reported AS intake (i.e., intake was greater than two standard deviations of the mean). The decision was made to not remove outliers in order to have a wide range of AS intake. Thus, these AS prediction equations may underestimate AS intake in those with extremely high AS intake. Although the plot did not reach the 95% agreement, no significant differences were detected in AS for the single-isotope equation, and it is important to note that slight variances are expected due to the different time periods covered by the two AS dietary assessment methods (isotope = habitual intake versus recall/record = recent intake). Conversely, a potential confound to the utility of the single-isotope model is the established energy intake underreporting in adults with overweight and obesity [31,32]. As such, reported AS intake may be underreported and, thus, the overestimation by the dual-isotope model may provide a better assessment of true reported AS intake in a population with overweight and obesity. Future studies using objective measures of energy intake should be conducted to examine this further.

Consistent with previous research in this area, these findings suggest that the dual-isotope method is not superior to the single-isotope method within a sample of adults living in southwest Virginia [9]. This single-isotope equation may only be valid in certain subsets of the population, and small validation studies may be required to develop population-specific prediction equations, especially for those with isotopically distinct diets (i.e., varied amounts of δ^13^C and δ^15^N dietary sources). For example, research conducted in Alaska with the Yup’ik population has demonstrated significantly improved predictions with the use of a δ^13^C and δ^15^N model (*R*^2^ = 0.33), while a model with δ^13^C value alone produced a lower *R*^2^ of 0.03 [10]. The difference in model fit may be attributed to diverse regional dietary consumption patterns, especially in regard to the consumption of marine animal protein that demonstrates a high δ^13^C relative to terrestrial protein sources [10]. Thus, by including δ^15^N, the δ^13^C contributed by marine animal protein consumption may be controlled for in the model. The use of a dual model may also prove useful in a post-weight loss population following a dietary pattern with higher fish consumption compared to terrestrial protein intake. Other confounds to the single-isotope model may be prevalent in populations with high consumption of livestock fed with corn-based diets vs. grass-fed, i.e., AS may not be causing the elevated δ^13^C values; thus, δ^15^N may be used as a correction factor [33]. The population living in the rural Appalachian region, which are represented in this investigation as well as our prior studies [7,8,9], has an established history of elevated AS and sugar-sweetened beverage consumption and increased risk of related co-morbidities [34]. Furthermore, this population may have higher error related to self-reported dietary intake and may benefit from objective measures of AS consumption [1]. Thus, validating an AS prediction equation for this high-risk population is an important target. Several rodent studies have demonstrated differences in δ^15^N values between red blood cells and plasma/serum [35,36], and correcting for this difference may provide further utility for the dual-isotope model, yet no differences have been demonstrated in human specimens [29]. Thus, due to the potential variation in δ^15^N values across specimen types and different isotopic diet profiles significantly impacting δ^13^C and δ^15^N values, dual-isotope models may be better predictors of AS intake in certain populations.

This study had several limitations that should be noted. The first was the reliance on self-reported dietary intake and the use of different assessment methods between the reference and test groups (i.e., dietary recalls vs. records). Using two types of assessment methods could be problematic due to the different sources of measurement error; thus, findings should be cautiously interpreted including if differences in predictive abilities exist between the single- vs. dual-isotope models and if this biomarker is able to accurately predict AS intake in those with high AS consumption levels [2]. Furthermore, objective measures of AS and energy intake, such as feeding studies or the use of doubly labeled water, should be used when developing future prediction models. It is important to note that the prediction of AS intake provided by the δ^13^C biomarker is a measure of habitual AS intake, and the turnover time of δ^13^C values correlate with the turnover time of the corresponding blood specimen type. δ^13^C values in the reference and test groups were collected at baseline, along with the first dietary recall/record. The following days of dietary intake were collected within one to two weeks post-δ^13^C collection. Thus, δ^13^C values obtained did not reflect the same time period as reported dietary intake. An additional limitation might arise from expected differences in turnover time between specimen samples, i.e., the use of whole blood isotope values to develop the prediction equations in the reference group and the use of serum and plasma isotope values in the test group. While red blood cells exhibit an average lifespan of 120 days [37], turnover times for the myriad individual proteins included in blood serum have not been determined. A previous large-scale study (*n* = 406) designed to test the agreement in δ^13^C and δ^15^N values among different blood components did not find significant differences when comparing serum to clot extracted from whole blood samples and stored without additives [30]. On a related note, various blood specimen types were pooled for this analysis; due to the fact of this, analysis of specific specimen types was not possible. It can be hypothesized that the single-isotope equation demonstrates the ability to closely predict AS intake regardless of blood specimen type; however, it is unknown if one blood specimen type over- or under- predicts compared to other specimen types. Finally, results should be interpreted with caution due to the significant differences in key demographics between the reference and test groups and the small sample size of the test group that included a wide range of inclusion criteria; yet, the consistency of the AS prediction equation across various demographic characteristics is promising.

## 5. Conclusions

This investigation explored the use of various blood specimen samples to predict AS intake by implementing previously developed prediction model equations into a test sample with significantly lower AS intake and different demographic characteristics. Because the ability to measure AS intake is needed in order to improve the ability to accurately assess associations between intake and health outcomes as well as to determine the impact that public policies have on reducing intake, the availability of the δ^13^C biomarker is promising. However, the lack of an objective measure of AS intake to validate the utility of δ^13^C and δ^15^N is limiting, and more research is needed to assess the biomarker with rigorous study designs such as controlled feeding. Future research should aim to test this prediction equation in children, assess diverse populations with varying AS and protein dietary sources, and determine associations between δ^13^C values and cardio-metabolic markers such as weight status, blood pressure, and glucose and lipid values. Future research is also needed to assess the utility of δ^15^N values individually and as part of a dual-isotope model.

## Figures and Tables

**Table 1:** Differences in participant demographics between the reference and test groups.

Characteristics	Reference Group (*n* = 256) *n* (%) Unless Otherwise Noted	Test Group (*n* = 56) *n* (%) Unless Otherwise Noted	Test Statistic and *p*-Value
Sex, *n* (%)			
Male	60 (23)	34 (61)	χ^2^ = 30.6
Female	197 (77)	22 (39)	*p* ≤ 0.001
Age (years), mean ± SD	42.4 ± 14.7	53.1 ± 16.0	F = 23.7
			*p* ≤ 0.001
Age category, *n* (%)			χ^2^ = 28.5 *p* ≤ 0.001
18–24 years	33 (13)	7 (12.5)	
25–44 years	117 (46)	7 (12.5)	
45–64 years	91 (35)	30 (53.5)	
≥65 years	16 (6)	12 (21.5)	
BMI (kg/m^2^), mean ± SD	31.8 ± 9.2	29.5 ± 4.1	F = 3.4 *p* = 0.07
BMI category, *n* (%)			χ^2^ = 10.0 *p* = 0.02
Underweight, ≤18.4	3 (1)	0 (0)	
Normal weight, 18.5–24.9	65 (25.5)	6 (11)	
Overweight, 25–29.9	65 (25.5)	24 (43)	
Obese, ≥30	124 (48)	26 (46)	
Added sugars intake (g), mean ± SD	88.8 ± 58.8	68.8 ± 43.4	F = 5.8 *p* = 0.02
δ^13^C (‰), mean ± SD	−19.1 ± 0.8	−19.5 ± 0.8	F = 10.3 *p* = 0.001
δ^15^N (‰), mean ± SD ^a^	7.4 ± 0.5	9.1 ± 0.3	F = 526.9 *p* ≤ 0.001

^a^ Sample size for δ^15^N for reference group = 115.

## Data Availability

The data presented in this study are available on request from the corresponding author. The data are not publicly available due to compilation of data from multiple previous studies.
